# The Modulating Effect of Cognitive Reserve on Dysfunctional Beliefs in Aging

**DOI:** 10.5964/ejop.17625

**Published:** 2026-02-27

**Authors:** Rosa Angela Fabio, Alessia Giordano

**Affiliations:** 1 Department of Biomedical and Dental Sciences and Morpho-Functional Imaging, University of Messina, Messina, Italy; 2 Department of Cognitive Science, University of Messina, Messina, Italy; Università Cattolica del Sacro Cuore, Milan, Italy

**Keywords:** cognitive reserve, dysfunctional thinking, cognitive flexibility, aging, cognitive performance

## Abstract

This study explores the role of cognitive reserve (CR) as a protective factor in late adulthood, focusing on its associations with cognitive performance, dysfunctional beliefs, and motivational orientation. A total of 100 older adults aged 65 to 93 completed standardized assessments: the Cognitive Reserve Index questionnaire (CRIq), the Montreal Cognitive Assessment (MoCA), and the Dysfunctional Beliefs Questionnaire (DBQ). Motivation was also assessed through qualitative responses categorized into thematic domains. Correlation analyses revealed that higher CR was significantly associated with better cognitive functioning (*r* = .62, *p* < .001) and fewer overall dysfunctional beliefs (*r* = -.26, *p* < .05), particularly self-criticism and frustration intolerance (both *r* = -.40, *p* < .01). No significant correlations were found with catastrophizing or absolute duty beliefs. Individuals with higher CR also showed a greater tendency toward positive motivational themes (χ^2^ = 7.98, *p* < .01), while those with lower CR more frequently reported negative motivations (χ^2^ = 5.55, *p* < .01). Structural equation modelling supported a model in which CR predicted cognitive performance, dysfunctional beliefs, and motivational orientation, with good overall fit (CFI = .97, TLI = .90, RMSEA = .08, SRMR = .03). Notably, CR had direct positive effects on MoCA scores (β = .62, *p* < .001) and positive motivation (β = .31, *p* < .01), and negative effects on dysfunctional beliefs (β = -.26, *p* < .05) and negative motivation (β = -.30, *p* < .05). These findings support the view that cognitive reserve contributes not only to cognitive resilience but also to more adaptive motivational and emotional patterns in aging. The results highlight the role of CR in enhancing cognitive performance and reducing maladaptive beliefs, suggesting a dynamic relationship between cognitive resources, emotional-motivational functioning, and individual differences in late adulthood.

Cognitive Reserve (CR) refers to the brain's adaptive capacity, functioning as a “brain power reserve”. It acts as a protective mechanism, enabling the brain to maintain optimal functioning despite neural damage or to develop new cognitive strategies when existing ones are compromised (Stern, 2013). The central concept of CR suggests that some brains function more efficiently than others, even when faced with similar levels of damage or neurodegeneration. This resilience is attributed to both innate and acquired brain characteristics (Kochhann et al., 2023).

At birth, brain sizes vary, with some individuals having larger brains than others, and each brain having its specific or general intellectual potentials (Stern et al., 2023). However, life experiences, education, occupation, nutrition, and overall health significantly impact the brain throughout life. These differences reflect the quality of internal connections and the overall health of the brain. A well-developed and nourished brain shows greater efficiency and resilience against both neurological diseases such as traumatic injuries, stroke, dementia and physical or psychological diseases such as diabetes, hypertension, or depression (Montine et al., 2019), while in childhood a cognitively stimulating environment fosters brain volume growth. Physical exercise and cognitive stimulation increase molecules that support brain plasticity, allowing the brain to adapt continually throughout life (Peng et al., 2022).

Intellectual endowments, education, experiences, and occupation significantly influence the brain's ability to compensate for damage and preserve its functions. Normal aging, associated with memory decline, executive function loss, and language impairment, progresses more slowly in individuals with higher levels of literacy (Gelfo, 2019). Cognitive Reserve Theory has led researchers to discover that a well-trained brain can use its resources more efficiently, recruiting additional brain areas when necessary (Ellison, 2021).

The concept of Brain Reserve is related but distinct, referring to structural brain resources like brain size, synapse count, or neuronal density that provide a passive buffer against neuropathology (Arenaza-Urquijo et al., 2015). While Brain Reserve reflects the brain’s quantitative capacity, Cognitive Reserve involves the active and flexible use of cognitive strategies to cope with brain changes (Stern, 2009).

Together, these reserves help explain why individuals with similar brain pathology may exhibit different clinical outcomes. Life-long cognitive stimulation, physical exercise, and enriched environments promote neuroplasticity, enhancing Cognitive Reserve (CR) and supporting better cognitive outcomes in aging and disease (Peng et al., 2022; Tucker & Stern, 2011). Individuals with higher CR cope better with neural damage than those with lower CR, even when matched for pathology and brain damage (Steffener & Stern, 2012). Thus, CR facilitates cognitive performance in brain disorders, acting as a dynamic, flexible, and “plastic” mechanism within the brain.

This view aligns with animal model data showing that enriched environments promote neurogenesis and increased expression of Brain-Derived Neurotrophic Factor (BDNF), a molecule essential for synaptic growth, remodelling, and overall brain plasticity (Brown & Ryan, 2003; Nithianantharajah & Hannan, 2006). These molecular adaptations support the brain’s ability to reorganize in response to injury or aging-related changes, strengthening neural networks and compensatory mechanisms involved in CR (Bosch et al., 2010). For example, living in an enriched environment appears to mitigate cognitive decline associated with Alzheimer’s disease (Tucker & Stern, 2011).

Longitudinal studies reveal the complex and heterogeneous trajectories of cognitive decline during aging. Montemurro et al. (2025), studying a cohort of older adults with cognitive difficulties, showed that individuals with higher cognitive reserve — estimated via education and occupational complexity — exhibited greater cognitive efficiency at baseline and maintained more stable cognitive performance over time compared to those with lower reserve. Importantly, even participants diagnosed with major neurocognitive disorders demonstrated a slower decline if they had higher cognitive reserve, suggesting that CR may slow cognitive deterioration despite ongoing brain pathology. Nyberg et al. (2020) reviewed data from the Betula prospective cohort study (Nilsson et al., 1997) and other longitudinal research, emphasizing the influence of genetic, biological, and lifestyle factors on individual differences in cognitive aging. Their work highlights the vital role of brain maintenance and compensatory mechanisms, shaped by both environmental and biological factors, which contribute to preserved cognitive function in some older adults. Together, these studies support a multifactorial and dynamic model of cognitive reserve, where neuroplasticity, molecular biology, and life experiences interact to shape the heterogeneity of cognitive aging trajectories.

Although significant advances have been made, the precise mechanisms and longitudinal effects of cognitive reserve remain active fields of investigation, warranting further research. In a recent review, Pettigrew and Soldan (2019) reported that while higher CR is linked to better cognitive performance, its impact on longitudinal cognitive trajectories remains unclear and warrants further research.

CR can be quantified through indirect indices such as education, professional achievements, and leisure activities. Literature provides evidence of a reduced risk of Alzheimer's in individuals with higher education levels (Allegri et al., 2010; Bernini et al., 2025; Panebianco et al., 2022, 2023; Scarmeas & Stern, 2004). Protective effects are also found in occupations involving high responsibility (Nucci et al., 2012; Wajman & Bertolucci, 2010) and leisure activities that involve learning, attention, memory, and other intellectual skills (Fabio et al., 2024; Velloso et al., 2025; Wilson et al., 2013). While these structural and experiential factors help build cognitive reserve, emerging evidence suggests that cognitive and emotional patterns, such as dysfunctional thinking, may also influence CR’s efficacy over time.

## Dysfunctional Thoughts and Their Impact on Cognitive Health

The relationship between dysfunctional thoughts and cognitive health has become increasingly relevant in the context of healthy aging and cognitive reserve (CR). While CR is typically described as a protective factor shaped by lifelong intellectual, occupational, and social engagement, recent evidence suggests that chronic psychological stress and maladaptive cognitive patterns may modulate its effectiveness (Oosterhuis et al., 2023; Porricelli et al., 2024).

Dysfunctional thoughts — such as catastrophizing, rumination, and cognitive rigidity — are maladaptive mental habits that distort reality and promote emotional distress (Beck, 1976). These thought patterns are strongly associated with mood and anxiety disorders and are known to intensify stress responses, reduce resilience, and negatively affect cognitive flexibility (Fabio & Caprì, 2023; Fabio, Caprì et al., 2021). Such psychological states may impair the brain's ability to scaffold new cognitive processes or activate compensatory mechanisms when neural efficiency declines.

The revised Scaffolding Theory of Aging and Cognition (STAC-r) highlights how negative psychological factors — such as depression or perceived stress — can interfere with the development and maintenance of compensatory neural scaffolds, thereby limiting the beneficial effects of CR (Oosterhuis et al., 2023). These mechanisms suggest that dysfunctional thoughts may not only reflect but actively diminish the protective effects of CR over time.

Importantly, cognitive-behavioral interventions have been shown to reduce maladaptive thoughts and improve emotional regulation, potentially supporting the preservation of CR by fostering more adaptive cognitive strategies (Beck, 1995; Gangemi et al., 2018). While previous research emphasized structural factors such as education and occupation, emerging data now point to the role of motivation, perceived control, and cognitive-emotional engagement as dynamic contributors to CR throughout the lifespan (Lavrencic et al., 2018; Savarimuthu & Ponniah, 2024).

This view encourages a more integrative approach in which CR is not only a predictor of cognitive aging outcomes, but also a modifiable construct influenced by psychological resilience and motivational orientation. For example, individuals with higher CR may be more inclined to pursue meaningful activities and engage in proactive stress regulation strategies, reinforcing their cognitive resilience across time.

Thus, while CR may function as a predictor in longitudinal models of aging, dysfunctional thinking patterns may act as moderators that impair or facilitate its expression, depending on the individual’s capacity for psychological adaptation. Future research should explore the interactions between CR, stress, and emotional-cognitive regulation to better understand interindividual differences in cognitive trajectories.

### Hypotheses

The general aim of this study is to investigate the role of cognitive reserve (CR) in modulating dysfunctional thinking among older adults. Drawing on the active model of CR and cognitive theories of emotional adaptation, we propose the following hypotheses:

#### Hypothesis 1

It is hypothesized that individuals with higher levels of cognitive reserve (CR) will demonstrate better cognitive performance on the Montreal Cognitive Assessment (MoCA), given that CR serves as a protective factor against age-related cognitive decline. CR is believed to reflect the brain’s ability to compensate for damage or decline, which could lead to more favorable outcomes on cognitive tests.

#### Hypothesis 2

Higher CR levels will be associated with fewer dysfunctional thoughts, including cognitive distortions such as self-criticism, catastrophizing, and frustration intolerance. These maladaptive thought patterns often exacerbate emotional distress and contribute to cognitive decline. CR is expected to mitigate these patterns by promoting more flexible and adaptive thinking.

#### Hypothesis 3

We hypothesize that higher CR would be associated with more positive motivational patterns, such as goal-directed behavior and a sense of meaning in life, and fewer negative motivational states, such as feelings of helplessness or disengagement. Given that CR is linked to better cognitive functioning and emotional regulation, individuals with higher CR are expected to exhibit more adaptive and proactive motivational attitudes.

Finally, although the cross-sectional design does not permit causal conclusions, we consider the theoretical possibility that persistent dysfunctional thinking may, over time, erode cognitive functioning and reduce resilience. This potential bidirectional relationship warrants exploration in future longitudinal research.

## Method

### Participants

The study included 100 Italian participants aged between 65 and 93 years (*M* = 81.0, *SD* = 7.33), comprising 54 females (54.0%) and 46 males (46.0%). Individuals with a diagnosis of dementia or other age-related neurodegenerative disorders were excluded. Participants were randomly selected based on their availability to participate in the study. Most participants completed all assessments independently; however, four individuals were assisted by a support partner (spouse or children) exclusively during the administration of the Cognitive Reserve Index questionnaire (CRIq). In terms of marital status, 72.0% were married or cohabiting, 17.0% widowed, and 1.0% divorced. Educational attainment was distributed as follows: 5.0% held a university degree, 40.0% completed secondary education, and 55.0% had only primary education. All participants provided written informed consent prior to participation.

A post hoc power analysis was conducted using G*Power 3.1 (Faul et al., 2009) to evaluate whether the sample size was sufficient to detect the effects observed in our main correlational analyses. With a total sample size of 100 participants and an alpha level of .05, the statistical power (1 – β) to detect a large effect (e.g., *r* = .62, the observed correlation between cognitive reserve and MoCA scores) was greater than .99. The power to detect a medium effect (*r* = -.40, observed between cognitive reserve and self-criticism) was approximately .90. These results suggest that the sample size was adequate for detecting moderate-to-large associations. However, the power to detect smaller effects, such as the correlation between cognitive reserve and overall dysfunctional beliefs (*r* = -.26), was lower (approximately .63), and this limitation is addressed in the Discussion section.

### Materials

All participants completed the following instruments:

#### Montreal Cognitive Assessment

The MoCA (Aiello et al., 2022; Nasreddine et al., 2005) is a brief cognitive screening tool designed to assess global cognitive functioning across multiple domains, including executive functions, attention, memory, language, visuospatial abilities, and orientation. The test consists of 30 items and typically requires 5 to 10 minutes for administration. Scores range from 0 to 30 (Nasreddin, et al., 2005). In this study, we administered the Italian version of the MoCA and corrected raw scores using the age- and education-adjusted norms developed by Aiello et al. (2022). These updated norms are based on a large sample of healthy Italian adults and allow for continuous correction of scores through regression-based formulas. Performance is expressed as an Equivalent Score (ES) ranging from 0 (Deficient performance) to 4 (Above-average performance), providing a standardized classification of cognitive status within the Italian population. All analyses in the present study were based on these corrected ES values rather than raw scores or fixed cut-offs from the original MoCA version. This approach ensures that demographic factors such as age and education level are appropriately accounted for in evaluating cognitive functioning.

#### Cognitive Reserve Index Questionnaire

The CRIq (Nucci et al., 2012) is a semi-structured interview developed to assess cognitive reserve based on an individual’s lifetime experiences in education, occupation, and leisure activities. It measures both the intensity and duration of engagement in cognitively stimulating activities. The CRIq generates a global cognitive reserve score, classified into five levels: Low (≤ 70), Medium-low (70–84), Medium (85–114), Medium-high (115–130), and High (≥ 130). Its psychometric properties have been validated in various studies, with an internal consistency coefficient of Cronbach’s alpha = 0.87 (Nucci et al., 2012).

#### Dysfunctional Beliefs Questionnaire

The DBQ (Fabio, Caprì et al., 2021) consists of 36 items designed to assess dysfunctional cognitive beliefs that contribute to cognitive distortions. The questionnaire evaluates four main domains: self-criticism and devaluation, catastrophizing, rigid demands, and frustration intolerance. Participants rate each item on a 5-point Likert scale, with higher scores reflecting stronger dysfunctional beliefs. The DBQ has demonstrated good internal consistency, with Cronbach’s alpha values ranging from 0.84 to 0.92 across subscales (Fabio, Caprì et al., 2021).

#### Motivation

Two semi-structured open-ended questions were used to collect qualitative and quantitative data on participants’ subjective perceptions of meaningful life experiences. These questions were adapted from Lawton et al. (2002): “What is the most important thing you have understood in this life?” and “How important is it to you on a scale from 1 to 10?” Participants’ verbal responses to the first question were transcribed and independently coded by two independent trained raters into five thematic categories:

- Positive (e.g., “That life is a gift and should be lived with gratitude”).
- Work-related (e.g., “That commitment to one’s job defines who you are”).
- Family-oriented (e.g., “That family always comes first, above everything else”).
- Negative (e.g., “That you cannot trust people and life is unfair”).
- Life-cycle philosophical (e.g., “That everything passes, and we must accept impermanence”).

The second question provided a quantitative rating of motivation importance, which was treated as a continuous variable in subsequent analyses.

### Procedure

Participants were initially contacted and invited to participate in the study. Upon consent, a researcher visited each participant’s home to conduct all assessments in a single session. After obtaining written informed consent, participants first completed the MoCA to assess cognitive status. Subsequently, the CRIq was administered, with some (four) participants receiving assistance from a support partner when needed. The DBQ and the motivation questions were administered last. The entire session lasted approximately 60 to 90 minutes. Data collection was conducted in a standardized manner by trained researchers to ensure consistency and reliability.

All procedures adhered to ethical standards of institutional, regional, and national research committees and were consistent with the 1964 Helsinki Declaration and its later amendments. Ethical approval was granted by the Ethics Committee for Psychological Research (Protocol Number 0034787, 23 October 2023).

### Statistical Analysis

All statistical analyses were conducted using IBM SPSS Statistics (Version 25) and AMOS (Version 19) for structural equation modeling (SEM). Descriptive statistics (means and standard deviations) were computed to summarize the main study variables, including cognitive reserve (CR), cognitive functioning, dysfunctional beliefs, and motivation.

Pearson’s product–moment correlations were used to examine the associations among CR, Montreal Cognitive Assessment (MoCA) scores, and dysfunctional beliefs. Significance levels were set at *p* < .05, *p* < .01, and *p* < .001.

To investigate differences in motivational orientation based on CR, a median split was used to categorize participants into high and low CR groups. Although the CRIq allows categorization into five CR levels (Nucci et al., 2012), to compare it with motivation orientation we treated the CRIq global score as a continuous variable to preserve data variability. Although dichotomizing continuous variables is often discouraged due to potential loss of information and power, recent evidence (Iacobucci et al., 2015) suggests median splits can be valid and parsimonious under certain conditions. Among participants categorized as “low CR” by our median split (scores ≤ 95), 38% corresponded to the “medium-low” level and 62% to the “medium” level according to Nucci et al.’s classification. Among participants classified as “high CR” (scores > 95), 58% belonged to the “medium”, 35% to the “medium-high”, and 7% to the “high” CR levels per the original scale.

Motivational responses were qualitatively coded into five thematic categories — positive, work-related, family-oriented, negative, and life-cycle philosophical motivations — and group differences were assessed using chi-square (χ^2^) tests of independence. An initial inter-rater agreement of 93% was achieved by two independent coders. Discrepancies between coders were discussed and resolved through consensus to ensure consistent categorization.

To further explore the hypothesized relationships among variables, structural equation modeling (SEM) with maximum likelihood estimation was performed. The model specified CR as an exogenous predictor of cognitive performance (MoCA), dysfunctional beliefs, and motivational orientation. Dysfunctional beliefs were also modeled as a mediator influencing both MoCA and motivational outcomes.

Model fit was evaluated using several goodness-of-fit indices: the chi-square statistic (χ^2^), the Comparative Fit Index (CFI), the Tucker-Lewis Index (TLI), the Root Mean Square Error of Approximation (RMSEA) with 90% confidence intervals, and the Standardized Root Mean Square Residual (SRMR). A good model fit was indicated by non-significant chi-square values (*p* > .05), CFI and TLI values ≥ .90, RMSEA ≤ .08, and SRMR ≤ .08 (Hu & Bentler, 1999).

All reported regression coefficients are standardized (β), and statistical significance was set at *p* < .05 for all path estimates. The final model demonstrated acceptable fit and supported the hypothesized direct and indirect associations among cognitive reserve, dysfunctional thinking, cognitive performance, and motivational patterns in older adults.

## Results

To test our hypotheses, we analysed the relationships between cognitive reserve (CR), dysfunctional thoughts, and cognitive performance. Table 1 presents the means and standard deviations for each variable considered.

**Table 1:** Descriptive Statistics (Means and Standard Deviations) for Key Measures

Measure	*M*	*SD*
Cognitive Reserve	95.18	16.58
CRI-School	101.91	16.31
CRI-Work	94.02	15.02
CRI-Leisure	92.18	22.62
MoCA	23.77	4.27
Visuospatial	2.59	1.23
Naming	2.60	0.82
Attention	4.28	1.70
Language	1.58	0.95
Abstraction	1.77	0.74
Memory	4.76	0.53
Orientation	5.66	0.92
Overall dysfunctional beliefs	43.26	16.98
Frustration Intolerance	11.68	4.83
Absolute Duty	13.92	6.44
Catastrophizing	9.00	6.43
Self-Criticism/Devaluation	8.23	5.55
Motivation	2.73	2.07

*Note*. MoCA scores have been corrected according to the Italian normative data by Aiello et al. (2022), which adjust raw scores based on age and education and provide Equivalent Scores (ES) ranging from 0 (Deficient performance) to 4 (Normal performance). The corrected mean (*M* = 23.77, *SD* = 4.33) reflects adjusted cognitive performance, and the ES (*M* = 2.59, *SD* = 1.23) was used in the main analyses. Higher values indicate better cognitive functioning.

### Correlation Analysis

Pearson’s correlation coefficients were computed to examine the relationships between cognitive reserve (CR), MoCA scores, and dysfunctional beliefs (see Table 2).

**Table 2:** Correlations Between Cognitive Reserve and Measured Variables

Variable	1	2	3	4	5	6	7
1. Cognitive Reserve (CR)	1	.65***	-.26*	-.42**	-.10	-.04	-.42**
2. MoCA		1	-.37**	-.35*	-.14	-.057	-.33*
3. Overall Dysfunctional Beliefs			1	.78***	.60***	.62***	.74***
4. Self-Criticism				1	.51***	.36**	.33**
5. Catastrophizing					1	.44***	.41***
6. Absolute Duty						1	.39***
7. Frustration Intolerance							1

*p < .05. **p < .01. ***p < .001.

As hypothesized, CR was positively correlated with cognitive performance as measured by the MoCA (*r* = .62, *p* < .001), indicating that individuals with higher cognitive reserve tend to perform better on cognitive assessments.

Supporting the second hypothesis, CR was inversely correlated with overall dysfunctional beliefs (*r* = –.26, *p* < .05), suggesting that individuals with greater cognitive reserve report fewer maladaptive thought patterns. When examining specific dysfunctional belief dimensions, CR showed a moderate negative correlation with self-criticism/devaluation (*r* = -.40, *p* < .01) and frustration intolerance (*r* = -.40, *p* < .01). Correlations with catastrophizing (*r* = -.10, *p* = .32) and absolute duty (*r* = -.04, *p* = .69) were not statistically significant.

### Motivational Differences Based on Cognitive Reserve

To examine the third hypothesis, we investigated whether cognitive reserve (CR) is associated with distinct motivational patterns in older adults. We hypothesized that individuals with higher CR would express more positive motivational orientations — such as goal-directed behavior and a sense of purpose — and fewer negative motivational states, such as helplessness or avoidance. Participants were divided into high and low CR groups using a median split. Motivational responses were qualitatively coded and categorized into thematic groups based on shared psychological functions and content. Specifically, five categories were identified:

1. **Positive Motivations** — Oriented toward personal growth, autonomy, or meaning.
*Example:* “I want to stay mentally active and continue learning.”
2. **Work-Related Motivations** — Linked to professional identity or a sense of contribution through labor.
*Example:* “I still want to feel useful through my volunteer work.”
3. **Family-Oriented Motivations** — Focused on caring for or being present for family members.
*Example:* “I want to be there for my grandchildren.”
4. **Negative Motivations** — Motivations primarily aimed at avoiding suffering or negative outcomes.
*Example:* “I just want to avoid pain and suffering.” or “I hope to avoid living through another war.”
5. **Life-Cycle Philosophy** – Abstract or existential reflections on the meaning of life stages.
*Example:* “Life is a cycle, and we must accept it as it comes.”

These categories allowed for both quantitative and qualitative analysis of motivational themes, facilitating comparison between individuals with different levels of CR. Table 3 shows the percentage distribution of different motivational themes reported by participants.

**Table 3:** Frequencies of Reported Motivational Categories

Motivation Type	Percentage (%)
Positive Motivations	31.0
Work-Related	4.0
Family-Oriented	51.0
Negative Motivations	12.0
Life-Cycle Philosophy	2.0

Chi-square analyses revealed significant differences between groups. Participants with higher CR were more likely to report positive motivations (χ^2^ = 7.98, *p* < .01), whereas those with lower CR were more likely to express negative motivations (χ^2^ = 5.55, *p* < .01). Notably, family-related motivations — despite being the most frequently reported — did not differ significantly by CR level, suggesting that familial meaning may be a shared motivational resource in later life regardless of cognitive reserve. These findings further support the role of CR as a protective factor not only for cognitive and emotional functioning, but also for sustaining motivational engagement in aging.

### Structural Equation Modeling

To further examine the relationships between cognitive reserve (CR), dysfunctional beliefs, cognitive performance, and motivational orientation, a path analysis was conducted using structural equation modeling (SEM). The model tested both direct and indirect pathways based on theoretical assumptions and previously observed correlations.

The proposed model included CR as a predictor of cognitive performance (MoCA scores), dysfunctional beliefs, and motivational orientation. Dysfunctional beliefs were also modeled as a potential mediator, influencing both MoCA scores and motivational orientation.

Model fit was assessed using standard indices: the chi-square statistic (χ^2^), the Comparative Fit Index (CFI), the Tucker-Lewis Index (TLI), the Root Mean Square Error of Approximation (RMSEA), and the Standardized Root Mean Square Residual (SRMR). Fit indices indicated an acceptable to good fit: χ^2^(2) = 5.63, p = .06; CFI = .97; TLI = .90; RMSEA = .08 [90% CI = .00–.17]; SRMR = .03.

**Figure 1:**
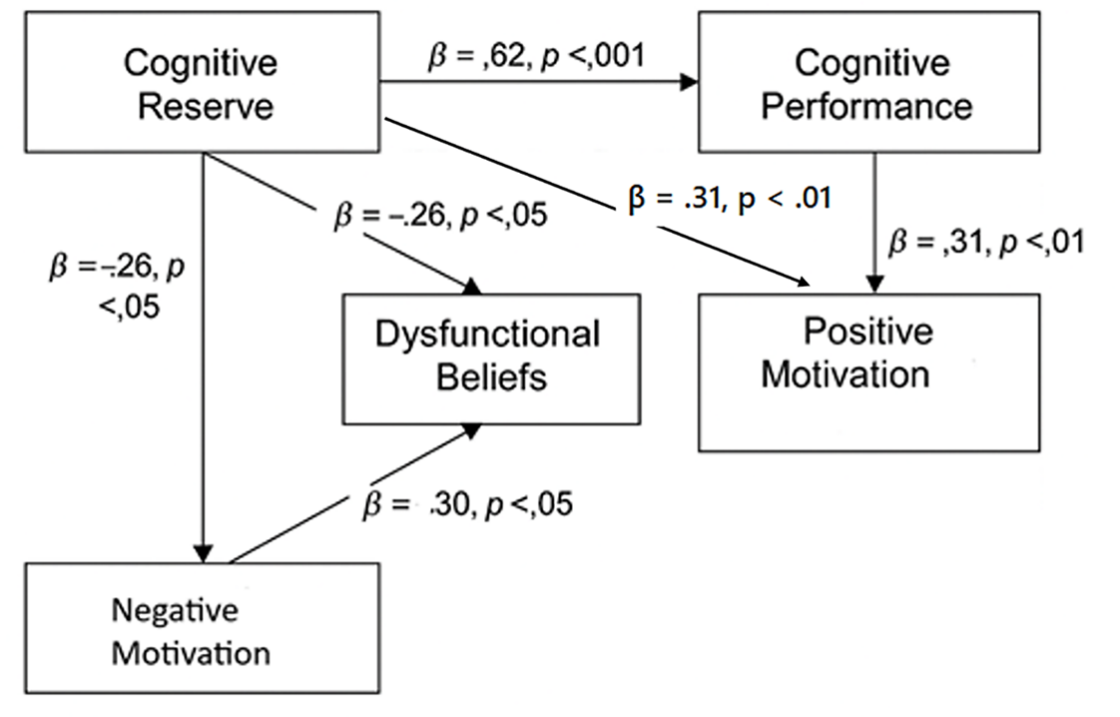
Path Diagram With Standardized Regression Coefficients Showing Direct and Indirect Effects of Cognitive Reserve on Cognitive Performance, Dysfunctional Beliefs, and Motivation *Note.* Model fit: χ^2^(2) = 5.63, *p* = .06; CFI = .97; TLI = .90; RMSEA = .08; SRMR = .03

Figure 1 displays the path diagram with standardized regression coefficients. Cognitive reserve had a strong direct effect on cognitive performance (β = .62, *p* < .001) and a moderate inverse effect on dysfunctional beliefs (β = -.26, *p* < .05) and negative motivation (β = -.30, *p* < .05). Additionally, CR showed a moderate direct positive effect on positive motivation (β = .31, *p* < .01), suggesting that higher CR is associated with more positive and goal-directed motivational states. Dysfunctional beliefs were negatively associated with MoCA scores (β = -.35, *p* < .01) and with positive motivation (β = -.30, *p* < .05).

## Discussion

The current study aimed to investigate the role of cognitive reserve (CR) in modulating dysfunctional thinking, cognitive performance, and motivational patterns in older adults. Our findings support the proposed hypotheses, suggesting that CR plays a significant role in protecting cognitive function and influencing emotional regulation and motivation, in line with previous research on the adaptive capacity of the brain (Kochhann et al., 2023; Stern, 2013). For this study, data, codebook and materials are available at Fabio (2025).

First, we observed a strong positive relationship between CR and cognitive performance, as measured by the Montreal Cognitive Assessment (MoCA). This supports Hypothesis 1, which proposed that individuals with higher CR would demonstrate better cognitive performance. The association between CR and MoCA is consistent with prior studies suggesting that CR enables the brain to compensate for damage, thus maintaining cognitive functions despite age-related decline (Stern et al., 2020, 2023). This relationship is further corroborated by Stern’s (2009) theory, which posits that individuals with higher CR actively engage compensatory mechanisms to manage cognitive challenges.

In line with Hypothesis 2, we also found that CR was negatively correlated with dysfunctional thoughts, specifically self-criticism and frustration intolerance. These results align with the theory that individuals with higher CR are better equipped to manage stress and negative emotional states (Fabio, Gallo & Colombo, 2021). Catastrophizing can erode emotional resilience and impede cognitive performance (Beck, 1976), particularly in older adults. Our findings suggest that cognitive reserve (CR) might act as a buffer against such distortions by promoting greater cognitive flexibility, metacognitive awareness, and adaptive emotion regulation. Individuals with higher CR may be better equipped to recognize and reframe irrational thoughts, rely on diverse problem-solving strategies, and engage neural compensatory mechanisms that help preserve cognitive functioning even in the presence of psychological stressors. Although the correlation with catastrophizing was not significant, the inverse relationship with self-criticism and frustration intolerance suggests that CR plays a protective role in managing maladaptive thought patterns that are known to exacerbate cognitive decline (Beheshti, 2025).

The third hypothesis, which posited that higher CR would be associated with more positive motivational patterns, was also supported by the data. Participants with higher CR expressed a greater orientation toward goal-directed behavior and a sense of purpose, whereas those with lower CR were more likely to report negative motivations. This finding is consistent with the work of Lawton et al. (2002), who emphasized the importance of motivation in cognitive aging. Higher levels of CR have been linked to more proactive engagement with life, which can enhance resilience and preserve cognitive health. In this study, motivational responses were categorized into thematic groups, and participants with higher CR were more likely to report positive motivations related to personal growth and meaning. These results highlight the role of CR not only in cognitive performance but also in fostering a sense of purpose and goal-directed behavior, both of which contribute to overall well-being in older adulthood.

Moreover, our path analysis provided a more comprehensive understanding of the relationships between CR, cognitive performance, dysfunctional thoughts, and motivation. The model demonstrated that CR had a direct and strong effect on cognitive performance and a moderate inverse effect on dysfunctional beliefs and negative motivation. These findings are consistent with Stern’s (2009) active reserve model, which suggests that individuals with higher CR are more likely to engage in compensatory strategies that protect cognitive and emotional functioning. The positive effect of CR on positive motivation further reinforces the idea that CR is associated with greater life engagement, emotional regulation, and the maintenance of cognitive performance across the lifespan.

Overall, the results emphasize the importance of interventions aimed at strengthening cognitive reserve (CR) through lifelong learning, cognitive training, physical exercise, and social engagement (Velloso et al., 2025). Additionally, research on enriched environments suggests that cognitive stimulation can promote neurogenesis and the release of brain-derived neurotrophic factor (BDNF), enhancing CR and protecting against neurodegenerative disease (Brown & Ryan, 2003). BDNF plays a crucial role in synaptic plasticity, supporting the formation and maintenance of neural circuits involved in memory, executive function, and attention (Bekinschtein et al., 2011; Park & Reuter-Lorenz, 2009). These neurobiological mechanisms — including synaptogenesis, dendritic remodelling, and increased network efficiency — are central to brain plasticity and may explain how individuals with higher CR can better compensate for neuropathological burden, thereby maintaining functional independence for longer (Stern et al., 2023).

### Limitations

While the results provide strong support for the hypotheses, several limitations should be considered. First, the cross-sectional design of the study limits the ability to draw causal conclusions. Longitudinal research would be valuable to better understand the directionality of the relationships between CR, dysfunctional thinking, and cognitive performance. Although the sample size was adequate for detecting moderate-to-large effects, the statistical power for smaller associations (e.g., between cognitive reserve and general dysfunctional beliefs) was more limited. This should be considered when interpreting non-significant findings.

Additionally, while the study examined a range of dysfunctional beliefs, the lack of significant findings for catastrophizing and absolute duty suggests that other cognitive patterns may be more relevant for understanding the impact of CR. Future studies could further investigate these dimensions of dysfunctional thinking and explore how they interact with CR in different contexts.

Longitudinal studies are needed also to determine whether changes in CR directly influence cognitive rigidity or if pre-existing dysfunctional beliefs contribute to cognitive decline over time. Additionally, the study relied on self-report measures, which may introduce response biases. Future research should integrate neuropsychological assessments and experimental paradigms to provide a more comprehensive understanding of these relationships.

### Conclusions and Future Directions

In conclusion, the findings of this study underscore the importance of cognitive reserve in promoting cognitive and emotional well-being in older adults. Higher CR appears to be associated with better cognitive performance, fewer dysfunctional thoughts, and more positive motivational patterns. These results highlight the potential for interventions aimed at enhancing CR — such as engaging in cognitively stimulating activities and promoting emotional resilience — to help preserve cognitive health and improve quality of life in aging populations.

Future research should continue to explore the complex interplay between CR, dysfunctional thinking, and motivational orientation to further elucidate the mechanisms that protect against cognitive decline and emotional distress in older adults. Specifically, prospective longitudinal studies and neurobiological investigations will be critical to deepen our understanding of how cognitive reserve modulates brain aging trajectories and resilience to psychological stressors.

Longitudinal studies are needed also to determine whether changes in CR directly influence cognitive rigidity or if pre-existing dysfunctional beliefs contribute to cognitive decline over time. Additionally, the study relied on self-report measures, which may introduce response biases. Future research should integrate neuropsychological assessments and experimental paradigms to provide a more comprehensive understanding of these relationships.

## Supplementary Materials

**Table:** 

Type of supplementary materials	Availability/Access
Data	
Cognitive reserve and dysfunctional thinking dataset.	Fabio (2025)
Code	
SPSS Syntax RreservaCognitiva code.	Fabio (2025)
Material	
No supplementary material available.	—
Study/Analysis preregistration	
The study was not preregistered.	—
Other	
RiservaCognitiva codebook.	Fabio (2025)

## Data Availability

For this article, the following Supplementary Materials are available: full data set, code, codebook.
